# Supplementary material for: A neural mechanism for detecting object motion during self-motion
Source: eLife. 2022 Jun 1;11:e74971. doi: 10.7554/eLife.74971 (PMC9159750; doi:10.7554/eLife.74971)
Supplement: Transparent reporting form [file elife-74971-transrepform1.docx]

***eLife’s* transparent reporting form**

We encourage authors to provide detailed information *within their submission* to facilitate the interpretation and replication of experiments. Authors can upload supporting documentation to indicate the use of appropriate reporting guidelines for health-related research (see [EQUATOR Network](http://www.equator-network.org/%20)), life science research (see the [BioSharing Information Resource](https://biosharing.org/)), or the [ARRIVE guidelines](http://www.plosbiology.org/article/info:doi/10.1371/journal.pbio.1000412) for reporting work involving animal research. Where applicable, authors should refer to any relevant reporting standards documents in this form.

If you have any questions, please consult our Journal Policies and/or contact us: [editorial@elifesciences.org](mailto:editorial@elifesciences.org).

**Sample-size estimation**

- You should state whether an appropriate sample size was computed when the study was being designed
- You should state the statistical method of sample size computation and any required assumptions
- If no explicit power analysis was used, you should describe how you decided what sample (replicate) size (number) to use

Please outline where this information can be found within the submission (e.g., sections or figure legends), or explain why this information doesn’t apply to your submission:

We collected more than one hundred neurons from two monkeys ( > 50 from each animal), which is a widely accepted sample-size in non-human primate research at the single-unit level. No explicit power analysis was uses. Results are largely consistent between the two animals.

**Replicates**

- You should report how often each experiment was performed
- You should include a definition of biological versus technical replication
- The data obtained should be provided and sufficient information should be provided to indicate the number of independent biological and/or technical replicates
- If you encountered any outliers, you should describe how these were handled
- Criteria for exclusion/inclusion of data should be clearly stated
- High-throughput sequence data should be uploaded before submission, with a private link for reviewers provided (these are available from both GEO and ArrayExpress)

Please outline where this information can be found within the submission (e.g., sections or figure legends), or explain why this information doesn’t apply to your submission:

Experiments (training sessions or physiological recording sessions) were performed on a daily basis during weekdays. We did not exclude any outliers. Some analyses require homogeneous experimental conditions (e.g., a fixed set of depth differences for population decoding analysis). In that case, we used a subset of a total dataset that meets such criterion. In all results, we unambiguously described the criterion and number of neurons used for analyses in the main text.

**Statistical reporting**

- Statistical analysis methods should be described and justified
- Raw data should be presented in figures whenever informative to do so (typically when N per group is less than 10)
- For each experiment, you should identify the statistical tests used, exact values of N, definitions of center, methods of multiple test correction, and dispersion and precision measures (e.g., mean, median, SD, SEM, confidence intervals; and, for the major substantive results, a measure of effect size (e.g., Pearson's r, Cohen's d)
- Report exact p-values wherever possible alongside the summary statistics and 95% confidence intervals. These should be reported for all key questions and not only when the p-value is less than 0.05.

Please outline where this information can be found within the submission (e.g., sections or figure legends), or explain why this information doesn’t apply to your submission:

We generally used non-parametric tests, as neural data often deviate from normality. To show population results, we used scatterplots that show all neurons included in the statistical tests. Thus, we have attempted to show the data in the rawest form that makes sense. Detailed statistics (e.g., mean, SD, confidence intervals) and tests used are given in the text or figure legends along with p-values. Exact p-values were reported using scientific notation.

(For large datasets, or papers with a very large number of statistical tests, you may upload a single table file with tests, Ns, etc., with reference to sections in the manuscript.)

**Group allocation**

- Indicate how samples were allocated into experimental groups (in the case of clinical studies, please specify allocation to treatment method); if randomization was used, please also state if restricted randomization was applied
- Indicate if masking was used during group allocation, data collection and/or data analysis

Please outline where this information can be found within the submission (e.g., sections or figure legends), or explain why this information doesn’t apply to your submission:

Trials with different experimental conditions were pseudo-randomly interleaved within a session, under computer control. Two monkeys used in the study experienced a mostly identical set of experimental conditions, except for a few datasets in the initial phase of the experiments. In most figures, data are grouped according to animal and presented for each animal separately. No other grouping was used.

**Additional data files (“source data”)**

- We encourage you to upload relevant additional data files, such as numerical data that are represented as a graph in a figure, or as a summary table
- Where provided, these should be in the most useful format, and they can be uploaded as “Source data” files linked to a main figure or table
- Include model definition files including the full list of parameters used
- Include code used for data analysis (e.g., R, MatLab)
- Avoid stating that data files are “available upon request”

Please indicate the figures or tables for which source data files have been provided:
